# ICT-Based Health Care Services for Individuals with Spinal Cord Injuries: A Feasibility Study

**DOI:** 10.3390/s20092491

**Published:** 2020-04-28

**Authors:** Wan-ho Jang, Seung-bok Lee, Dong-wan Kim, Yun-hwan Lee, Yun-jeong Uhm, Seung-wan Yang, Jeong-hyun Kim, Jong-bae Kim

**Affiliations:** 1 Department of Occupational Therapy, The Graduate School, Yonsei University, Wonju 26493, Korea; wh.jang@yonsei.ac.kr (W.-h.J.); dwan3303@naver.com (D.-w.K.); enn1210@naver.com (Y.-h.L.); 2 Clinical Team, Yonsei Enabling Science and Technology Research Center, Wonju 26493, Korea; supermandrlee@gmail.com (S.-b.L.); shilover0@gmail.com (S.-w.Y.); 3 Department of Ergonomic Therapy, The Graduate School of Health and Environment, Yonsei University, Wonju 26493, Korea; uyjot@naver.com; 4 Usability Center, Yonsei Enabling Science Technology Research Center, Wonju 26493, Korea; otrehab486@gmail.com; 5 Department of Occupational Therapy, College of Health Science, Yonsei University, Wonju 26493, Korea

**Keywords:** ICT, home health care service, spinal cord injury, occupational therapy

## Abstract

In the Republic of Korea, 90.5% of those living with spinal cord injury (SCI) are faced with medical complications that require chronic care. Some of the more common ones include urinary tract infections, pressure sores, and pain symptomatology. These and other morbidities have been recognized to deteriorate the individual’s health, eventually restricting their community participation. Telerehabilitation, using information and communication technology, has propelled a modern-day movement in providing comprehensive medical services to patients who have difficulty in mobilizing themselves to medical care facilities. This study aims to verify the effectiveness of health care and management in the SCI population by providing ICT-based health care services. We visited eight individuals living with chronic SCI in the community, and provided ICT-based health management services. After using respiratory and urinary care devices with the provision of home visit occupational therapy, data acquisition was achieved and subsequently entered into a smart device. The entered information was readily accessible to the necessary clinicians and researchers. The clients were notified if there were any concerning results from the acquired data. Subsequently, they were advised to follow up with their providers for any immediate medical care requirements. Digital hand-bike ergometers and specialized seating system cushions are currently in development. The ICT-based health care management service for individuals with SCI resulted in a favorable expected level of outcome. Based on the results of this study, we have proposed and are now in preparation for a randomized clinical trial.

## 1. Introduction

Individuals with spinal cord injury (SCI) have impaired motor and sensory function, which results in difficulty with the performance of daily activities and social participation [1,2]. Many are confronted with medical complications upon returning to their respective individual homes in the community, following discharge from rehabilitation facilities [3]. Pressure ulcers may occur as a result of sensory impairment, and pulmonary related morbidities arise because of respiratory muscle function deterioration [4]. These and other related complications can lead to difficulties in activity and participation, and in severe cases, may result in death [5]. Co-morbidities inevitably lead to an array of unfortunate losses: physically, emotionally, socially, and economically [6]. In the Republic of Korea, 90.5% of those with SCI have recently reported medical complications stemming from their underlying SCI [7]. The most common ones included cystitis, pressure sores, and pain. The majority of these individuals also report that the complications are a detriment to their overall well-being. Therefore, it is essential to prevent and systematically manage these complications. Traditionally, medical complications are regularly managed by physicians in a medical facility setting. However, many with SCI have difficulty accessing proper medical services, primarily due to the profound inconvenience from transportation barriers and from the economic burdens resulting from hospital expenses [8]. To help overcome these barriers, health care providers can offer services through the latest innovative communications technologies, otherwise known as telehealth [9]. Telerehabilitation, using information and communication technology (ICT), refers to providing comprehensive medical services to patients who have difficulty accessing medical facilities or who prefer a home-setting for undergoing rehabilitation [10]. Technological advances such as these enable patients to actively engage in their care, especially when confronted by challenges when accessing appropriate medical services [11]. Recently, a health care service model via a remote digital management system has been implemented into clinical practice. This study aims to verify the effectiveness of the health management model in providing an ICT-based health care system services for individuals with SCI.

## 2. ICT-Based Health Care Services for Individuals with SCI

**Figure 1:**
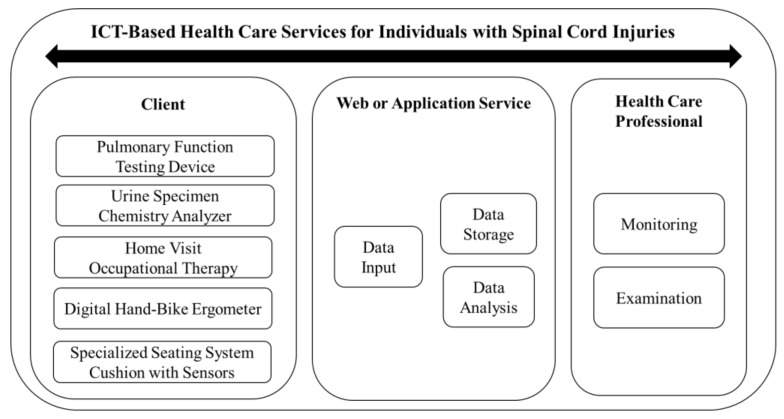
Model of information and communication technology (ICT)-based health care services.

Based on our literature review of previous research studies, a model for the management of individuals with SCI was constructed with the implementation of three of the five proposed interventions. Licensed occupational therapists visited their homes and used ICT devices. After using respiratory and urinary home-care devices with the provision of home visit occupational therapy, data acquisition was achieved and subsequently uploaded onto a server using a smart device. The entered information was readily accessible to the appropriate clinicians and researchers. Clients were notified of any concerning results shown from the acquired data (e.g., urinalysis indicating possible treatment). Subsequently, patients were advised to follow-up in the clinic with their providers for any indicated and immediately necessary medical care. Other ICT equipment including digital hand-bike ergometers and specialized seating system cushions with sensors are currently in the development phase (Figure 1).

### 2.1. Pulmonary Function Testing Device

The hand-held device measures forced expiratory volume in one second (FEV1) and peak expiratory flow (PEF), which are typical indicators of respiratory function [12,13]. The quantifiable breathing ability can be measured directly by the patient. Occupational therapists will visit homes to train and assist with measurements, as needed. This data will be stored on the server. If the doctor or occupational therapist determines that the client has a problem with a pulmonary function, the client will be prompted to seek medical attention at the hospital.

### 2.2. Urine Specimen Chemistry Analyzer

A simple bed-side model urine chemistry analyzer is used to screen a total of 10 components detectable in an amply collected specimen, including glucose, protein, nitrites, and white blood cells. The urine sample will be examined on site with the aid of an occupational therapist visiting the home. Data are stored on the server and can be accessed by clinicians and researchers. If an outlier is noted on urinalysis, the client will be referred for a detailed examination and possible therapeutic intervention.

### 2.3. Home Visit Occupational Therapy

The occupational therapist visits the home and provides occupational therapy to the client. Therapy consists of interventions and training in the following areas: daily living activities, home environment modifications, self-exercise training, range of motion exercises along with assistive technology, and community service information. The resultant data from the evaluation and treatment are entered into the smart device and the data are subsequently stored in the server.

### 2.4. Digital Hand-Bike Ergometer

There is a scarcity of properly adapted exercise equipment available for individuals with cervical level spinal cord injury. A hand-bike ergometer system is currently undergoing development for appropriate use for these individuals. Previous studies have reported that hand-bikes for individuals with SCI help to improve motor function and overall health [14]. This particular equipment associated with the “home exercise training” component of the model was excluded in this pilot study.

### 2.5. Specialized Seating System Cushion with Sensors

Pressure ulcers in SCI are one of the most common symptoms, and can become extremely severe complications [15]. The underlying reason for the initial breakdown of skin integrity is attributed to sensory paralysis, especially of the lower limbs. An appropriate seating system serves as a long term preventive intervention. We are currently in the development phase of a customized cushion with pressure sensors that can monitor the pressure in real-time. The ultimate goal in using this device is to prevent sores by detecting pressure applied to those areas where ulcers develop. This cushion was excluded from the current study.

### 2.6. Web or App Service of Health Management 

**Figure 2:**
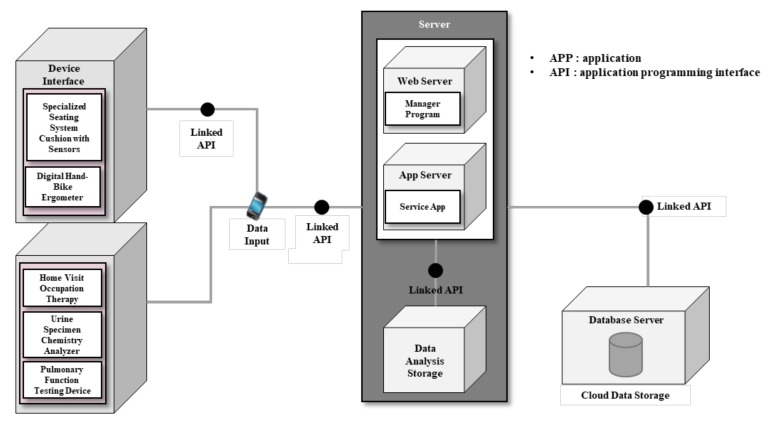
System concept diagram.

Data acquired following the application of five interventions are entered by the therapist or patient, and subsequently stored on the server in association with the application programming interface (API; Figure 2). There are notably two main servers—the web server and app server—involved as the main players in this study. The “web server” serves as the communication channel between doctors and therapists. Therapists will enter acquired data into the server following the onsite home intervention. They consult clients’ status with doctors, while doctors monitor the entered data. The app server allows the study participants to access their recorded individual health information and schedule. Under circumstances in which maintaining the privacy of sensitive information is required, encryption and the separate storage of data offer further added security.

**Figure 3:**
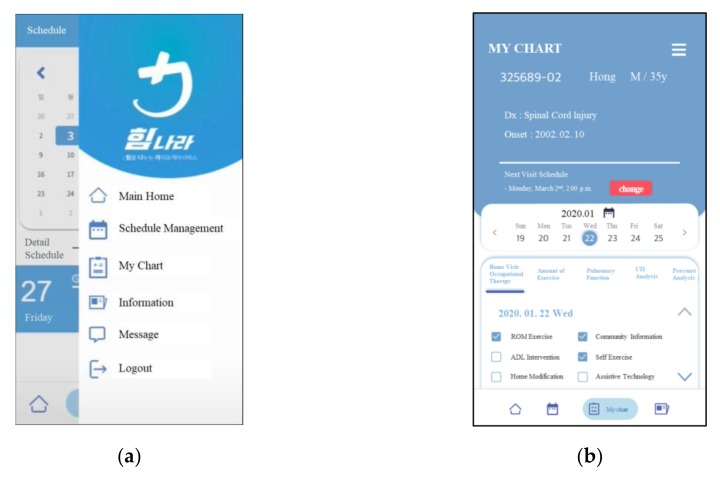
App (application) service of health care management: (**a**) The app is running and (**b**) the user can verify all of the scheduled activities.

ICT-based applications (Figure 3) were developed with two groups in mind—study participants and visiting therapists—resulting in two versions of the app. The differentiating feature of the participant’s version is that their access is limited to only their own file. Participants logged into the app can typically monitor their health information and also schedule their next visit with the therapist. Participants can also send and receive messages via the app directly to therapists or doctors. The second version is customized for the home visiting therapists. The application allows them access to all of the entered information of the participants to whom they have been assigned. Direct communication with participants and physicians using the app is also feasible. Data input is allowed while home visits are in progress or up until the end of the following day. Physicians can communicate any resultant data with therapists, while therapists can communicate with physicians for any consultative advice while the home visit service is in progress.

## 3. Study

### 3.1. Target Selection Criteria

The inclusion criteria for selecting participants were as follows: (1) Those with underlying SCI who are 20 years of age or older, (2) those who are able to agree to the study participation in writing, (3) those who are able to understand and follow verbal instructions, and (4) those who agree to participate in the entire study. The exclusion criteria were those who have: (1) Current symptoms of urinary tract infection that require hospitalization, such as systemic fever and an elevated white blood cell count; or (2) those who have had pressure sores that require current wound care management. 

### 3.2. Study Design and Outcome Measurements

The design of this study is a mixed research method of combined quantitative study. The ICT-based interventions were conducted for four months. We plan to measure two respiratory indicators using the pulmonary function testing device, and three health related indicators using three assessment tools. 

The outcome measurements are as follows:

- FEV1 and PEF (forced expiratory volume in one second and peak expiratory flow, respectively)
- SCIM (spinal cord independence measure)
- WHOQOL-BREF (brief version of the World Health Organization quality of life scale)
- ESES (exercise self-efficacy scale)

All of the participants were individually assessed by the respective visiting therapists before and after evaluation, using the above variables.

FEV1 and PEF are frequently used indicators for assessing respiratory function. Each indicator was measured three times, followed by recording the highest output value.

SCIM is a tool for assessing the levels of activities of daily living (ADL) in individuals with SCI, and has three sub-divisions, namely: (1) self-care, (2) respiration and sphincter management, and (3) mobility. The total maximum score is 100 points, with higher scores indicating independent levels of ADL.

WHOQOL-BREF assesses 26 items divided into four domains (physical health, psychological, social relationships, and environment). Each item consists of a five-point Likert scale, ranging from 26 to 130 points. Higher scores translate into a higher quality of life.

The ESES assessment has a total of 18 items. Each item is a scale consisting of 10 points up to 100 points. Higher scores are reflective of higher self-efficacy in exercise. In the data analysis, the average score was calculated by adding the scores of each question.

### 3.3. Statistical Analysis

Statistical analysis was completed using SPSS version 21 software. The general characteristics of the participants are presented as mean and standard deviation. Pre-post evaluations are also presented as mean and standard deviation. Statistical tests were performed using the Wilcoxon Signed Rank Test. A nonparametric test was used, owing to a small sample size (*n* = 8). The significance level was 0.05.

## 4. Results

### 4.1. Participants and Intervention

Based on our selection criteria, we recruited participants from the Seoul and Wonju areas of South Korea with assistance from the Korea Spinal Cord Injury Association and Wonju Severance Hospital of Yonsei University, respectively. A total of eight recruits were enrolled and participated in this study. Their characteristics are shown in Table 1. All of the subjects were initially evaluated in the Department of Physical Medicine and Rehabilitation prior to initiating the interventions, and consented to their full participation in the study. Licensed occupational therapists, after training in the application of the ICT model interventions, visited participants’ homes bi-weekly, from October 2019 to January 2020, to perform ICT-based interventions and assessment.

### 4.2. Outcomes

There was improvement in all five areas of the outcome indicators. However, only one (*p* = 0.018) of the five outcomes was significantly different (Table 2).

The FEV1 and PEF of the pulmonary function testing increased after the intervention. However, it was not statistically significant (*p* = 0.161). In a preliminary interview, most participants expected to have improved breathing function after using the respiratory device concomitantly with breathing exercises. Many expressed satisfaction with their participation in the interventional activities, as one participant remarked, “I am glad to check the breathing function periodically by using the ICT devices” (J., 47 years old).

The SCIM results for the assessment of ADL showed a significant increase following intervention (*p* = 0.018). Occupational therapists assisted in achieving functional improvement by ADL training. The participants and their respective care givers were satisfied after receiving training on the proper use of ADL-related assistive devices, such as portable lifts, ramps, and wheelchairs. Some have expressed eagerness to install safety handle bars to further promote independence in toilet use. However, the cost burden was a deterrence, yet gain, to uphold the ongoing challenges posed upon these individuals.

The quality of life scores by WHOQOL-BREF improved, but were not significantly different (*p* = 0.483). Nevertheless, participants reported high levels of satisfaction by an overall increased quality of life from their participation and in receiving the services rendered. One participant commented, “After one treatment, I noted pain relief and improvement in my overall movement; I am satisfied and look very much forward to the next treatment session” (L., 45 years old). Another participant, after being routinely assessed by request, was found to have proteinuria. The visiting occupational therapist intervened by referral to urology, and appropriate management and input were rendered. The fact that a clinician can remotely analyze their bladder status with respect to potentially underlying infection or other morbidity via the results of a urinary chemical analyzer adds reassurance and has a profound impact on patients.

The self-efficacy for exercise performance measured with ESES was higher post intervention, but was not significant (*p* = 0.310). Most respondents felt a subjective need for an exercise regimen. However, they did not know how to routinely and properly engage in a fitness program. Upon initiation of the ICT-based intervention, many expressed satisfaction from having received guided instructions on a home exercise program. A participant remarked, “Now I use the thera-band to do a variety of exercises, regularly, in the convenience of my home” (K., 52 years old).

Five occupational therapists carried out the specific task of using the devices involved in the delivery of the ICT model healthcare. To enhance the reliability of results, therapists received appropriate training in the standardize application of these devices, including the spirometer for pulmonary function assessment and a chemical analyzer for urinalysis. Prior to the implementation of the model using these devices, its feasibility was piloted on five individuals with spinal cord injury, in which the trained therapists carried out the standardized tasks. As the needs and functions of each individual with spinal cord injury can vary, we expected similarly that appreciable variations may result in the actual delivery of the standardized interventions.

## 5. Discussion

Traditional healthcare services are provided by medical professionals via direct face-to-face contact between patients and providers in limited and unsuitable spaces [16]. The rapidly developing ICT-based medical care is a promising new model of a healthcare service delivery system, with an unprecedented method of use. The use of various self-management devices in chronic diseases enables the user to monitor health status in real time outside of specialty care clinics [17]. It reflects the transformation of generalized and standardized medicine to personalized medicine, suitable to meet the individual needs [18,19]. This study was focused on people with spinal cord injuries, thus making it necessary to have different health indicators for the varying types of disability that stem from the underlying spinal cord injuries. Occupational therapists and physicians both had access to and management of the stored data when using the ICT devices. Furthermore, clinicians monitored patients’ health information and allowed for the recognition of any concerning clinical risk indicators in advance. Further research will aid in identifying precise indicators that will aid in the timely delivery of clinical interventions. Although we did not observe a significant level of improvement in these areas following intervention in this study, the investigating clinicians believe that it is valuable information and is appropriate to monitor and confirm.

Individuals with spinal cord injuries have reduced pulmonary function because of respiratory muscle paralysis. Monitoring and assessment are necessary, because compromised respiratory function can ultimately lead to death. Prior studies have emphasized the importance of monitoring respiratory function in people with spinal cord injuries [20,21,22]. We used FEV1 and PEF as indicators to evaluate the clients’ respiratory function. These two indicators are common variables used in the measurement of pulmonary function [23,24,25]. Portable respirators for measuring these indicators have been evaluated as safe and appropriate for people with spinal cord injuries [26]. The study by McDonald (2018)—using portable smart devices for ten days in seven individuals with spinal cord injury—reported that there was no difficulty or adverse events when using the device. Furthermore, signs and symptoms, such as cardiac arrhythmia and stiffness, were not observed, and thereby safely ensured data acquisition. Herein, the results of the respiratory function improved, but there was no significant difference [26]. In other studies that showed a significant increase, investigators enhanced the intervention methods by adding respiratory or abdominal muscle resistive training [27,28]. The results from our study will serve to be useful in future research directions, as the pulmonary function was not shown to have weakened and it reflected the advantage of periodic monitoring using the ICT devices.

The consideration of user and organizational perspectives of ICT medical services is salient at this juncture [29,30]. Many interventions with ICT devices for the users focus on their activities and experiences [31,32,33,34,35]. It has also been our experience, from previous studies related to our ICT devices, that clients’ levels of satisfaction and usability were reported to be high [36]. We measured post-interventional levels of change in (1) ADL, (2) exercise awareness, and (3) quality of life in this study, within the scope of the subjective values placed upon their individual lives in the setting of their respective homes. All scores were slightly higher than prior to intervention, with the scores from daily activities significantly increased. Enhancing the performance of the meaningful and purposeful activities of clients is an important goal of occupational therapy [37,38]. These activities boost self-esteem and inevitably add value to the lives of individuals [39].

The ICT model provided a basis for improving their daily lives by effectively promoting independence, adaptation, and participation in the community. In order to implement a systematic health management system for people with spinal cord injuries, we plan to apply sensor-based pressure sore prevention cushions and aerobic exercise equipment in our subsequent research endeavors. The ICT-based medical service model has promising potential to improve the efficiency of the current health care system. It can reduce unnecessary expenditures in transportation costs, manage chronic diseases, promote wellness, resolve blind spots in medicine, and magnify the value of client-centered healthcare [40]. In order to generate such benefits, it is necessary to accumulate, exchange, and use various types of medical information in a scholarly manner within the research community. Moreover, it is incumbent on us to accomplish this judiciously, so as to prevent the leakage of personal information, which apparently is a growing social problem according to researchers [41,42]. Thus, the scientific community is reminded that policies pertaining to the processing and protection of personal health information should be kept in the highest confidence, and are essential for the activation of ICT in the field of health care delivery.

## 6. Conclusions 

The ICT-based health care service model for community-dwelling SCI individuals presented in this study shows the feasibility of improving their overall health and well-being. Both occupational therapists and physicians can access and manage stored data when using the ICT devices. Physicians can monitor patients’ health information and allow for the clinical recognition of any concerning risk indicators in advance. Although we did not observe a significant level of improvement in the tested areas following intervention in this study, clinicians believe that it is valuable information and is appropriate to monitor. Based on the results of this pilot study, we recognized that further investigation is warranted in order to identify the precise indicators that will aid in the timely delivery of clinical interventions. We hypothesize that this type of health care service model could promote quality of life among individuals with spinal cord injury by targeting the prevention of secondary medical complications. Our expectation is that the results of the RCT will influence policy making and will ultimately be implemented into the health care insurance system. 

### Limitations

Five occupational therapists individually carried out the interventional tasks with subjective assessment, thereby the introduction of researchers’ bias is undeniable. This is especially emphasized as variations were expected, given varying characteristics with respect to the subjective needs and functional levels of the individual participants. The effectiveness of the model should be verified by stratification according to levels of function. The sample size and absence of a control group was a limiting factor in this pilot study. A larger study population with matched controls for comparative studies is necessary in order to verify the clinical effectiveness. The short study period was disadvantageous; a six month follow up period would be more appropriate in our future study. The delivery of interventions occurring in two geographic locations may limit the generalization of the findings. In the future, a broader expansion of areas with the involvement of large tertiary care centers may serve to be highly advantageous.

## Figures and Tables

**Table 1:** General characteristics of participants.

Characteristic (*n* = 8)	M ± SD or *n*
Age (yr)	56.88 ± 6.33
Sex (male)	6
(female)	2
Onset duration (year)	18 ± 12
Level of injury	
Tetraplegia	4
Paraplegia	4
Completeness of injury	
Complete	7
Incomplete	1
Cause of injury	
Traffic accident	3
Fall down	3
Industrial accident	1
Disease	1

**Table 2:** Results of Pre and Post testing.

Outcome Measures	Pre-Test	Post-Test	*z*	*p*
**FEV1 (L/sec)**	1.73 ± 0.68	1.84 ± 0.68	1.402	0.161
**PEF (L/min)**	344.75 ± 171.13	369.21 ± 161.33	1.400	0.161
**SCIM**	31.13 ± 14.19	35.63 ± 18.31*	2.375	0.018
**WHOQOL-BREF**	72.00 ± 16.64	75.50 ± 19.35	0.701	0.483
**ESES**	40.42 ± 23.03	53.96 ± 21.06	1.014	0.310

Note. Values are presented as mean ± standard deviation or as otherwise indicated. Abbreviations: FEV1, forced expiratory volume in one second; PEF, peak expiratory flow; SCIM, spinal cord independence measure, WHOQOL-BREF; brief version of the World Health Organization quality of life scale; ESES, exercise self-efficacy scale.* Significantly different from the pre-value (*p* < 0.05).

## References

[1] Liem N.R., McColl M.A., King W., Smith K.M. (2004). Aging with a spinal cord injury: Factors associated with the need for more help with activities of daily living. Arch. Phys. Med. Rehabil..

[2] Fonseca L., Tigra W., Navarro B., Guiraud D., Fattal C., Bo A., Fachin-Martins E., Leynaert V., Gelis A., Azevedo-Coste C. (2019). Assisted Grasping in Individuals with Tetraplegia: Improving Control through Residual Muscle Contraction and Movement. Sensors.

[3] Amatachaya S., Wannapakhe J., Arrayawichanon P., Siritarathiwat W., Wattanapun P. (2011). Functional abilities, incidences of complications and falls of patients with spinal cord injury 6 months after discharge. Spinal Cord.

[4] Sezer N., Akkuş S., Uğurlu F.G. (2015). Chronic complications of spinal cord injury. World J. Orthop..

[5] McKinley W.O., Jackson A.B., Cardenas D.D., Michael J. (1999). Long-term medical complications after traumatic spinal cord injury: A regional model systems analysis. Arch. Phys. Med. Rehabil..

[6] Krueger H., Noonan V.K., Trenaman L.M., Joshi P., Rivers C.S. (2013). The economic burden of traumatic spinal cord injury in Canada. Chronic Dis. Inj. Can..

[7] Korea Spinal Cord Injury Association. www.kscia.org/board/view/menu03_05/21450.

[8] Hossain M.S., Harvey L.A., Rahman M.A., Bowden J.L., Islam M.S., Taylor V., Muldoon S., Herbert R.D. (2017). A pilot randomised trial of community-based care following discharge from hospital with a recent spinal cord injury in Bangladesh. Clin. Rehabil..

[9] Lai B., Rimmer J., Barstow B., Jovanov E., Bickel C.S. (2016). Teleexercise for persons with spinal cord injury: A mixed-methods feasibility case series. JMIR Rehabil. Assist. Technol..

[10] Wellbeloved-Stone C.A., Weppner J.L., Valdez R.S. (2016). A systematic review of telerehabilitation and mHealth interventions for spinal cord injury. Curr. Phys. Med. Rehabil. Rep..

[11] Akhtar R., Alam S., Siddiquee N.K.A. (2019). Telemedicine: An ICT based healthcare approach to ensure health service for all. Int. J. Community Med. Public Health.

[12] Zhou P., Yang L., Huang Y.X. (2019). A smart phone based handheld wireless spirometer with functions and precision comparable to laboratory spirometers. Sensors.

[13] Tiftik T., Gökkaya N.K.O., Malas F.U., Tunç H., Yalçın S., Ekiz T., Erden T., Akkuş S. (2015). Does locomotor training improve pulmonary function in patients with spinal cord injury?. Spinal Cord.

[14] Kim D.I., Lee H., Lee B.S., Kim J., Jeon J.Y. (2015). Effects of a 6-week indoor hand-bike exercise program on health and fitness levels in people with spinal cord injury: A randomized controlled trial study. Arch. Phys. Med. Rehabil..

[15] Byrne D.W., Salzberg C.A. (1996). Major risk factors for pressure ulcers in the spinal cord disabled: A literature review. Spinal Cord.

[16] Zakaria N., Affendi S., Zakaria N., Dwivedi Y.K., Khoumbati K., Lal B., Srivastava A. (2010). Managing ICT in healthcare organization: Culture, challenges, and issues of technology adoption and implementation. Handbook of Research on Advances in Health Informatics and Electronic Healthcare Applications: Global Adoption and Impact of Information Communication Technologies.

[17] Elliott T.R., Brossart D., Berry J.W., Fine P.R. (2008). Problem-solving training via videoconferencing for family caregivers of persons with spinal cord injuries: A randomized controlled trial. Behav. Res. Ther..

[18] Patsakis C., Venanzio R., Bellavista P., Solanas A., Bouroche M. Personalized medical services using smart cities’ infrastructures. Proceedings of the IEEE International Symposium on Medical Measurements and Applications (MeMeA).

[19] Fengou M.A., Mantas G., Lymberopoulos D., Komninos N., Fengos S., Lazarou N. (2012). A new framework architecture for next generation e-health services. IEEE J. Biomed. Health Inform..

[20] Berlly M., Shem K. (2007). Respiratory management during the first five days after spinal cord injury. J. Spinal Cord Med..

[21] Casha S., Christie S. (2011). A systematic review of intensive cardiopulmonary management after spinal cord injury. J. Neurotrauma.

[22] Ryken T.C., Hurlbert R.J., Hadley M.N., Aarabi B., Dhall S.S., Gelb D.E., Rozzelle C.J., Theodore N., Walters B.C. (2013). The acute cardiopulmonary management of patients with cervical spinal cord injuries. Neurosurgery.

[23] McLachlan A.J., McLean A.N., Allan D.B., Gollee H. (2013). Changes in pulmonary function measures following a passive abdominal functional electrical stimulation training program. J. Spinal Cord Med..

[24] Cornwell P., Ward E., Lim Y., Wadsworth B. (2014). Impact of an abdominal binder on speech outcomes in people with tetraplegic spinal cord injury: Perceptual and acoustic measures. Top. Spinal Cord Inj. Rehabil..

[25] Mueller G., de Groot S., van der Woude L., Hopman M.T. (2008). Time-courses of lung function and respiratory muscle pressure generating capacity after spinal cord injury: A prospective cohort study. J. Rehabil Med..

[26] McDonald T., Stiller K. (2019). Inspiratory muscle training is feasible and safe for patients with acute spinal cord injury. J. Spinal Cord Med..

[27] Roth E.J., Stenson K.W., Powley S., Oken J., Primack S., Nussbaum S.B., Berkowitz M. (2010). Expiratory muscle training in spinal cord injury: A randomized controlled trial. Arch. Phys. Med. Rehabil..

[28] Postma K., Haisma J.A., Hopman M.T., Bergen M.P., Stam H.J., Bussmann J.B. (2014). Resistive inspiratory muscle training in people with spinal cord injury during inpatient rehabilitation: A randomized controlled trial. Phys. Ther..

[29] Shin D., Hwang Y. (2017). Integrated acceptance and sustainability evaluation of Internet of Medical Things. Internet Res..

[30] Shin D.H., Biocca F. (2017). Health experience model of personal informatics: The case of a quantified self. Comput. Hum. Behav..

[31] Hoffmann T., Cantoni N. (2008). Occupational therapy services for adult neurological clients in Queensland and therapists’ use of telehealth to provide services. Aust. Occup. Ther. J..

[32] Hoenig H., Sanford J.A., Butterfield T., Griffiths P.C. (2006). Development of a teletechnology protocol for in-home rehabilitation. J. Rehabil. Res. Dev. Clin. Suppl..

[33] Shin D.H. (2017). Conceptualizing and measuring quality of experience of the internet of things: Exploring how quality is perceived by users. Inform. Manag..

[34] Shin D.H., Biocca F. (2017). Explicating user behavior toward multi-screen adoption and diffusion. Internet Res..

[35] Shin D.H., Lee S., Hwang Y. (2017). How do credibility and utility play in the user experience of health informatics services?. Comput. Hum. Behav..

[36] Jang W., Kim D., Kim J., Yang S., Uhm Y., Kim J. ICT-Based Health Care Services for People with Spinal Cord Injury: A Pilot Study. Proceedings of the 17th International Conference on Smart Homes and Health Telematics (ICOST).

[37] Trombly C.A. (1995). Occupation: Purposefulness and meaningfulness as therapeutic mechanisms. Am. J. Occup. Ther..

[38] Crabtree J. (2000). What is a worthy goal of occupational therapy?. Occup. Ther. Health Care.

[39] Ryan P., Kobb R., Hilsen P. (2003). Making the right connection: Matching patients to technology. Telemed. J. E Health.

[40] Abaidoo B., Larweh B.T. (2014). Consumer health informatics: The application of ICT in improving patient-provider Partnership for a Better Health Care. Online J. Public Health Inform..

[41] Haluza D., Jungwirth D. (2015). ICT and the future of health care: Aspects of health promotion. Int. J. Med. Inform..

[42] Olanrewaju R.F., Ali N.A., Khalifa O., Manaf A.A. (2013). ICT in telemedicine: Conquering privacy and security issues in health care services. Electron. J. Comput. Sci. Inform. Technol..

